# Somatostatin receptor 2 (SSTR2) expression is associated with better clinical outcome and prognosis in rectal neuroendocrine tumors

**DOI:** 10.1038/s41598-024-54599-4

**Published:** 2024-02-19

**Authors:** Joo Young Kim, Jisup Kim, Yong-il Kim, Dong-Hoon Yang, Changhoon Yoo, In Ja Park, Baek-Yeol Ryoo, Jin-Sook Ryu, Seung-Mo Hong

**Affiliations:** 1 Department of Pathology, Chung-Ang University Hospital, College of Medicine, Chung-Ang University, Seoul, Republic of Korea; 2 Department of Pathology, Gil Medical Center, Gachon University College of Medicine, Inchon, Republic of Korea; 3 Department of Nuclear Medicine, Asan Medical Center, University of Ulsan College of Medicine, Seoul, Republic of Korea; 4 Departments of Gastroenterology, Asan Medical Center, University of Ulsan College of Medicine, Seoul, Republic of Korea; 5 Departments of Oncology, Asan Medical Center, University of Ulsan College of Medicine, Seoul, Republic of Korea; 6 Departments of Colon and Rectal Surgery, Asan Medical Center, University of Ulsan College of Medicine, Seoul, Republic of Korea; 7 Department of Pathology, Asan Medical Center, University of Ulsan College of Medicine, Seoul, Republic of Korea

**Keywords:** Somatostatin, Receptor 2, Rectum, Neuroendocrine tumor, Prognosis, Biotechnology, Cancer, Gastroenterology, Medical research

## Abstract

Somatostatin analogues have recently been used as therapeutic targets for metastatic or surgically unresectable gastroenteropancreatic (GEP) neuroendocrine tumors (NETs), and associated somatostatin receptor (SSTR) expression has been well demonstrated in most GEP NETs, with the exception of rectal NETs. SSTR2 immunohistochemical expressions were evaluated in 350 surgically or endoscopically resected rectal NETs and compared to clinicopathologic factors. SSTR2 expression was observed in 234 (66.9%) rectal NET cases and associated tumors with smaller size (p = 0.001), low pT classification (p = 0.030), low AJCC tumor stage (p = 0.012), and absence of chromogranin expression (p = 0.009). Patients with rectal NET and SSTR2 expression had significantly better overall survival than those without SSTR2 expression both by univariable (p = 0.006) and multivariable (p = 0.014) analyses. In summary, approximately two-thirds of rectal NETs expressed SSTR2. SSTR2 expression was significantly associated with favorable behavior and good overall survival in patients with rectal NETs. Furthermore, SSTR2 expression can be used as prognostic factors. When metastatic disease occurs, SSTR2 expression can be used a possible target for somatostatin analogues.

## Introduction

Rectal neuroendocrine tumors (NETs) are becoming more common among gastroenteropancreatic (GEP) NETs, owing to improved screening quality with colonoscopy^1–3^. They account for the majority of GEP-NETs in the Korea, Japan, United States, and Taiwan^1–3^. Despite the fact that L-cell type rectal NETs were considered to have uncertain malignant potential in the 2010 World Health Organization (WHO) grading system, all rectal NETs are now classified as malignant according to the 2019 WHO grading scheme^4,5^. This change may indicate that a subset of L-cell type rectal NETs exhibit malignant characteristics, such as lymph node or distant metastasis^6–8^. As a result, novel prognostic factors and therapeutic targets for metastatic or surgically unresectable rectal NETs are required.

Somatostatin (SST) is a peptide hormone that binds to somatostatin receptors (SSTRs) and which can be found in many organs, including the pancreas, central nervous system, and gastrointestinal tract^9–12^. It inhibits endocrine and exocrine secretion, as well as angiogenesis and cellular proliferation^13^. The physiologic functions of SST are mediated by interactions with SSTRs. SSTRs have five subtypes of G-protein-coupled transmembrane receptors (SSTR1–5) that mediate different biologic function of SST^14–16^. SSTR2 is the most frequently expressed subtypes in both GEP-NETs and also in normal tissue^17^. However, the frequency and expression pattern are different according to tumor types, location and patient characteristics^18^.

Somatostatin analogues (SSAs), such as octreotide and lanreotide, have been used to treat GEP NETs^19,20^. By binding to SSTR, SSAs provide symptomatic relief by inhibiting hormone hypersecretion and cellular proliferation of NETs^21^. In addition, radiolabeled SSAs are used to find the location and staging of NETs by SSTR-targeting scintigraphy or positron emission tomography (PET)/computed tomography (CT)^21,22^. SSTR expression, particularly SSTR2 and SSTR5 expression, has been used as a surrogate marker of peptide receptor radionuclide therapy (PRRT) for NENs^23–25^. SSTR2 expression, in particular, has been shown to be useful in several GEPNENs, with their clinicopathologic correlation, prognostic significance and utility of SSAs as therapeutic tools^26,27^. In the previous studies, most rectal NETs, particularly those of small size (≤ 1 cm), showed a favorable prognosis^6,7,28^. Complete endoscopic or surgical removal of these lesions is thus curative in the absence of additional PRRT or radiologic diagnostic tools. However, a small proportion of rectal NETs have showed aggressive behavior, such as advanced pT classification, presence of lymphovascular and perineural invasion, lymph node metastasis and even distant metastasis^6^. Thus, there is need for evaluating SSTR expression status for additional treatment or diagnostic modality in a subset of advanced or aggressive rectal NETs. SSAs currently available have a high affinity for SSTR2A, and immunohistochemical expression of SSTR2 in pancreatic NETs was linked to therapeutic effect of SSAs^29,30^. However, the association of SSTR2 expression status with clinicopathologic significances in rectal NETs with a large cohort has not been evaluated.

In this study, we evaluated SSTR2 immunohistochemical expression in rectal NETs and correlated with clinicopathologic factors, including patients’ survival.

## Materials and methods

### Case collection

After approval from the Institutional Review Board (approval number: 2014-0580) with a waiver of patients’ consent, a total of 350 surgically or endoscopically resected rectal NETs) between 2000 and 2014 were selected from Asan Medical Center, Seoul, Republic of Korea. Clinical data, such as patients’ age, sex, clinical procedure and survival outcome, were reviewed from electronic medical records. The institutional review board of Asan Medical Center, Seoul, Republic of Korea approved this study with waiver of informed consent (approval number: 2014-0580). All procedures were performed in accordance with the 1964 Declaration of Helsinki and its later amendments or comparable ethical standards.

### Pathologic study

Pathologic data, including tumor size, location, depth of invasion, mitotic count, Ki-67 labeling index, lymphovascular and perineural invasion, lymph node and distant metastasis, resection marginal status and immunohistochemical results of synaptophysin and chromogranin expression, was reviewed. Cases were classified as NET grade 1 and grade 2 according to 2019 WHO classification on the basis of mitotic count and Ki-67 labeling index^5^. The TNM stage was classified based on the 8th American Joint Committee on Cancer (AJCC) cancer staging manual^31^.

### Tissue microarray and immunohistochemistry

All hematoxylin and eosin slides of each case were reviewed and representative slides and paraffin blocks were retrieved. Tissue microarrays (TMAs) were constructed form representative paraffin blocks with tissue microarrayer (Uni TMA Co Ltd, Seoul, Republic of Korea). One 2.0 mm core was punched from donor tumor blocks and replaced into recipient blocks. Immunohistochemical staining was performed as previously described^6^. Briefly, from each TMA block, 4 μm thick sections were cut and deparaffinized and hydrated in xylene and ethanol. Endogenous peroxidase was blocked and heat-induced antigen retrieval was done. Sections were incubated with primary rabbit monoclonal antibody for SSTR2 (1:6400, ab134152, Abcam, Cambridge, UK).An OptiView DAB Detection Kit (Ventana Medical Systems) was used for the brown chromogen of SSTR2. Slides were counterstained with hematoxylin and dehydrated with ethanol. The result of SSTR2 immunohistochemical staining was graded into 4 groups on the basis of the extent of membranous staining (0, ≤ 5%; 1+, 6–25%; 2+, 26–50%; 3+, 51–75%; 4+, ≥ 76%). The cases were reclassified to 0 as negative group and 1+, 2+, and 3+ as positive groups as previously described^6,29^.

### Statistical analyses

Chi-squared test and Fisher’s exact test were performed to analysis the association between SSTR2 expression and clinicopathologic factors. The overall and recurrence free survival was evaluated with the Kaplan–Meier analysis with the log-rank test. The prognostic significance of SSTR2 was evaluated using the Cox proportional hazards regression model. P value < 0.05 was considered statistically significant. All statistical analyses were performed using SPSS version 18.0 (SPSS Inc., Chicago, IL, USA).

## Results

### Clinicopathologic characteristics of rectal NETs

The characteristics of rectal NETs are summarized in Table 1. The mean age was 48.5 ± 11.4 years (range 22–77 years) with a male to female ratio of 1.1:1. The median follow up period was 66.5 months (range 1–213 months). There were 324 (92.6%) NET grade 1 and 26 (7.4%) NET grade 2 cases. No grade 3 NET was included. The mean tumor size was 0.6 ± 0.4 cm (range 0.1–3.5 cm). When the NET size was dichotomized, sizes 1 cm or smaller were observed in 316 cases (90.3%) and sizes larger than 1 cm were noted in 34 cases (9.7%). The number of mitosis in 2 mm^2^ was < 2 in 335 (95.7%) and ≥ 2 in 15 (4.3%) cases. Seven cases received surgical resection and 343 cases were endoscopically resected. Proper muscle invasion was identified in 3 (42.9%) cases. Endoscopically resected rectal NETs (343 cases) were not applicable for proper muscle invasion and lymph node metastasis, as endoscopically resected specimens contained mucosa and submucosa only. Lymphovascular and perineural invasion was identified in 8 (2.3%) and 4 (1.1%) cases, respectively. Lymph node and metachronous distant metastasis was observed in 3 (42.9%), and 1 (0.3%) cases, respectively. According to the AJCC staging, there were 341 (97.4%) pT1, 7 pT2 (2.0%), 1 pT3 (0.3%), and 1 pT4 (0.3%) tumors, respectively and 338 (96.6%) stage I, 5 (1.4%) stage II, 5 (1.4%) stage III, and 2 (0.6%) stage IV cases, respectively. The median follow-up period was 67 months (range 1–214 months).

**Table 1:** SSTR2a expression and correlation with clinicopathologic factors of rectal neuroendocrine tumors.

Clinicopathologic factors	N (%)	SSTR2 expression		
		Absent (%)	Present (%)	*p* value
Age (years)				0.325
≤ 50	200 (57.1)	62 (31.0)	138 (69.0)	
> 50	150 (42.8)	54 (36.0)	96 (64.0)	
Sex				0.645
Male	187 (53.4)	64 (34.2)	123 (65.8)	
Female	163 (46.6)	52 (31.9)	111 (68.1)	
Grade				0.868
G1	324 (92.6)	107 (33.0)	217 (67.0)	
G2	26 (7.4)	9 (34.6)	17 (65.4)	
Size				0.001*
≤ 1 cm	316 (90.3)	96 (30.4)	220 (69.6)	
> 1 cm	34 (9.7)	20 (58.8)	14 (41.2)	
Proper muscle invasion (n = 7)^a^				0.809
Absent	4 (57.1)	3 (75.0)	1 (25.0)	
Present	3 (42.9)	2 (66.7)	1 (33.3)	
Lymphovascular invasion				0.306
Absent	342 (97.7)	112 (32.7)	230 (67.3)	
Present	8 (2.3)	4 (50.0)	4 (50.0)	
Perineural invasion				0.074
Absent	346 (98.9)	113 (32.7)	233 (67.3)	
Present	4 (1.1)	3 (75.0)	1 (25.0)	
Lymph node metastasis (n = 7)^a^				0.147
Absent	4 (57.1)	2 (50.0)	2 (50.0)	
Present	3 (42.9)	3 (100.0)	0 (0.0)	
Distant metastasis				0.155
Absent	349 (99.7)	115 (33.0)	234 (67.0)	
Present	1 (0.3)	1 (100.0)	0 (0.0)	
pT classification				0.030*
pT1	341 (97.4)	110 (32.3)	231 (67.7)	
pT2-4	9 (2.6)	6 (66.7)	3 (33.3)	
AJCC stage group				0.012*
I	338 (96.6)	108 (32.0)	230 (68.0)	
II–IV	12 (3.4)	8 (66.7)	4 (33.3)	
Chromogranin expression				0.009*
Absent	289 (82.6)	87 (30.1)	202 (69.9)	
Present	61 (17.4)	29 (47.5)	32 (52.5)	
Somatostatin expression				0.070
Absent	324 (92.6)	105 (32.4)	219 (67.6)	
Present	19 (5.4)	10 (52.6)	9 (47.4)	

*SSTR* somatostatin receptor, *AJCC* American Joint Committee on Cancer.

*Statistically significant at *p* < 0.05.

^a^Cases of endodscopically resected neuroendocrine tumors were not applicable for proper muscle invasion and lymph node metastasis and were excluded from this analysis.

### SSTR2 expression in rectal NETs

**Figure 1:**
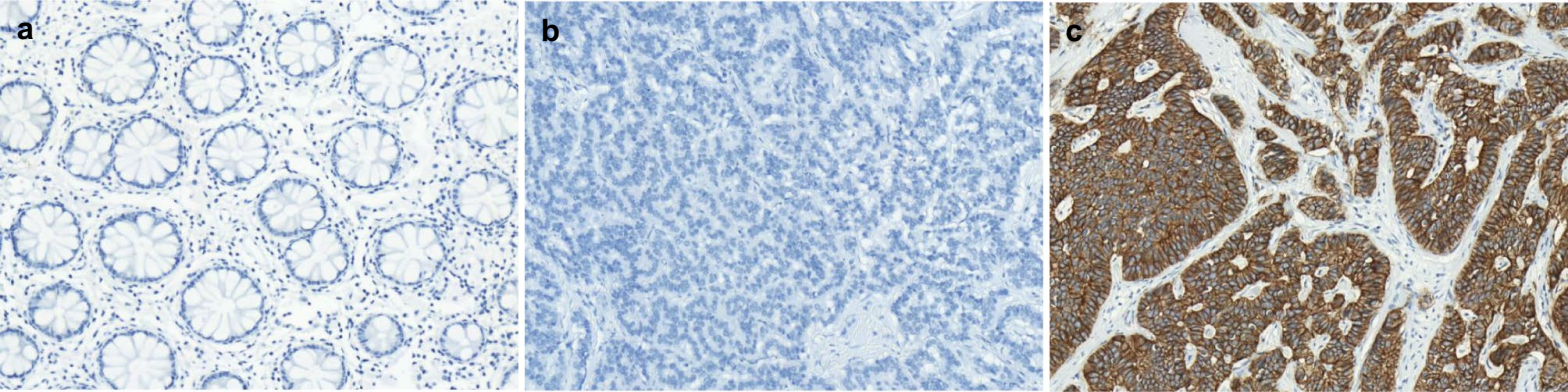
Representative images of SSTR2 immunohistochemical staining. (**a**) Normal rectal mucosa does not show SSTR2 expression. (**b**) Negative and (**c**) positive SSTR2 expression in rectal neuroendocrine tumors.

**Figure 2:**
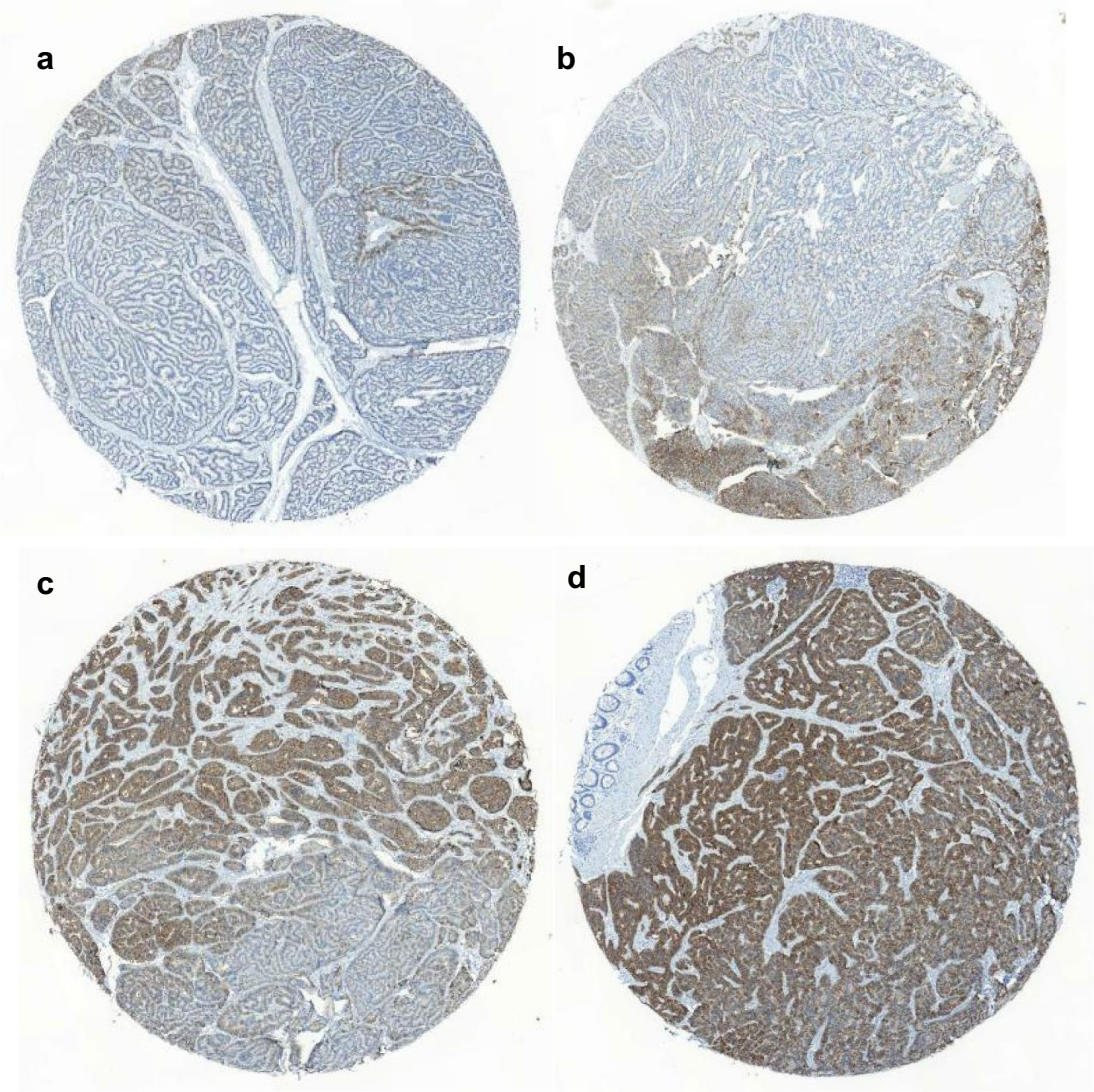
Representative images of rectal neuroendocrine tumors based on the SSTR2 immunohistochemical grading. SSTR2 immunohistochemical (**a**) grade 1, (**b**) grade 2, (**c**) grade 3, and (**d**) grade 4.

Representative images of SSTR2 expression in normal rectal mucosa and rectal NETs are illustrated in Figs. 1 and 2. Peritumoral non-neoplastic colonic mucosa was evaluated in 83 cases and none of them showed SSTR2 expression. In contrast, SSTR2 expression was observed in 234 (66.9%) rectal NETs. SSTR2 expression was significantly associated with small tumor size (p = 0.001), low pT classification (p = 0.030), low AJCC stage (p = 0.012), and absence of chromogranin expression (p = 0.009; Table 1).

### Survival analyses according to SSTR2 expression in rectal NETs

**Figure 3:**
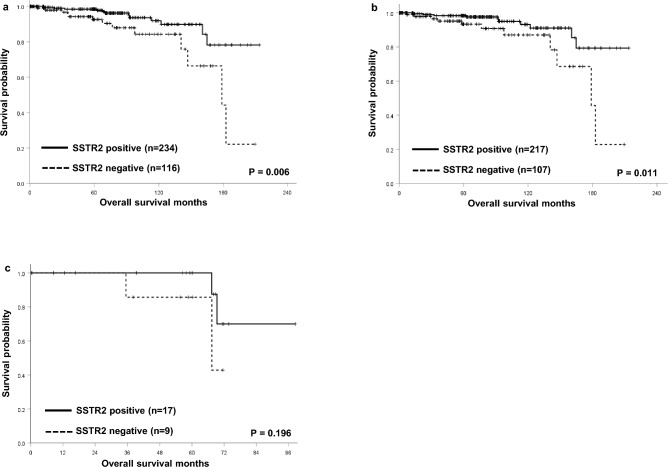
Kaplan–Meier survival analyses of rectal neuroendocrine tumors. (**a**) The overall 5-year survival rate of patients with SSTR2 expression was significantly better than those without expression (98.5% vs. 92.6%, p = 0.006). (**b**) Patients with grade 1 rectal NET and SSTR2 expression had significant better overall 5-year survival rate than those without SSTR2 expression (98.4% vs. 93.4%, p = 0.011). (**c**) The overall 5-year survival rate of grade 2 rectal NET patients with SSTR2 expression was better than those without SSTR2 expression though statistically not significant (100.0% vs. 85.7%, p = 0.196).

The overall survival of rectal NET patients with SSTR2 expression was significantly better than those without SSTR2 expression [hazard ration (HR) 0.346; 95% confidential interval (CI) 0.157–0.759; p = 0.006]. The 5-year survival rate of rectal NET patients with SSTR2 expression was significantly better than those without SSTR2 expression (98.5% vs. 92.6%, p = 0.006; Fig. 3a). Subgroup analysis based on tumor grade showed that grade 1 rectal NET patients with SSTR2 expression had significant better overall survival than those without SSTR2 expression (5-year survival rate, 98.4% vs. 93.4%, p = 0.011; Fig. 3b). In contrast, the overall 5-year survival rate of grade 2 rectal NET patients with SSTR2 expression was better than those without SSTR2 expression though statistically not significant (100.0% vs. 85.7%, p = 0.196; Fig. 3c).

### Univariable and multivariable survival analysis

Clinicopathologic factors including low grade (HR 5.719, CI 1.793–18.243, p = 0.001), absence of lymphovascular invasion (HR 4.455, CI 1.027–19.317, p = 0.029), absence of lymph node metastasis (HR 6.371, CI 0.826–49.136, p = 0.041), and absence of distant metastasis (HR 72.812, CI 8.132–651.909, p < 0.001) were significantly correlated with better overall survival in rectal NET patients by univariable analyses (Table 2).

**Table 2:** Univariate and multivariate analyses.

Clinicopathologic factors	5YSR (%)	Univariate analysis			Multivariate analysis		
		HR	95% CI	*p* value	HR	95% CI	*p* value
Age (years)				0.054			
≤ 50	96.6	1.00					
> 50	96.9	2.167	0.967–4.856				
Sex				0.784			
Male	98.0	1.00					
Female	95.4	1.116	0.507–2.456				
Grade				0.001*			0.002*
NET G1	96.9	1.00			1.00		
NET G2	95.2	5.719	1.793–18.243		6.633	2.023–21.746	
Size				0.685			
≤ 1 cm	96.8	1.00					
> 1 cm	96.2	1.354	0.311–5.889				
Proper muscle invasion				0.232			
Absent or NA^a^	96.7	1.00					
Present	75.0	3.210	0.424–24.296				
Lymphovascular invasion				0.029*			0.492
Absent	97.0	1.00			1.00		
Present	87.5	4.455	1.027–19.317		1.789	0.341–9.390	
Perineural invasion				0.668			
Absent	96.7	1.00					
Present	#						
Lymph node metastasis				0.041*			0.633
Absent or NA^a^	96.7	1.00			1.00		
Present	50.0	6.371	0.826–49.136		0.542	0.044–6.702	
Distant metastasis				< 0.001*			< 0.001*
Absent	97.1	1.00			1.00		
Present	0	72.812	8.132–651.909		58.519	6.116–559.950	
pT classification				0.388			
pT1	96.6	1.00					
pT2–4	80.0	2.374	0.314–17.942				
AJCC stage group				0.058			
I	97.0	1.00					
II–IV	90.9	3.775	0.862–16.537				
Chromogranin expression				0.900			
Absent	96.0	1.00					
Present	96.3	0.934	0.320–2.726				
SSTR2 expression				0.006*			0.014*
Absent	92.6	1.00			1.00		
Present	98.5	0.346	0.157–0.759		0.365	0.164–0.815	

*Statistically significant at *p* < 0.05.

^a^Endoscopically resected neuroendocrine tumors were regarded as not applicable cases.

^#^All cases are censored so statistics are not calculated.

*SSTR* somatostatin receptor, *NA* not applicable, *AJCC* American Joint Committee on Cancer, *HR* hazard ratio, *CI* confidence interval.

Multivariable analyses were conducted with factors that were shown by the univariable analyses to be significant (Table 3). SSTR2 expression (p = 0.014), low tumor grade (p = 0.002), and absence of distant metastasis (p < 0.001) were an independent prognostic factors in rectal NET patients (Table 2).

## Discussion

SSAs have been used to treat NETs, and radiolabeled SSAs have been increasingly used for imaging and therapy^31–34^. The expression of SSTR in NETs is the rationale for these clinical applications^35^. Among five subtypes of the SSTRs, SSTR2 is the most commonly expressed subtype in the NETs^36–38^. SSTR2 expression has previously been reported in GEP NETs, but it has not been specifically reported in rectal NETs. To the best of our knowledge, this is the first study on SSTR2 expression and its clinicopathologic correlation in large cohort of rectal NETs, including patients’ survival.

SSTR2 expression was identified in approximately 70% of rectal NETs in the present study. Previous studies demonstrated that SSTR2 expression in a small cohort of rectal NETs (range 3–13 patients) and the majority of them were included as a component of left colon or colorectal NET cohorts^18,38–41^. The proportion of SSTR2 expression in rectal NETs ranged from 14 to 100% in the previous studies^18,38,39,41^. The prevalence (70%) of SSTR2 expression in rectal NETs in the present study is similar with that of Oana et al., and they reported 53.8% (7 of 13 cases) of rectal NETs with SSTR2 expression^39^. Previously reported prevalence of SSTR2 expression in rectal NETs is variable^18,38,39,41^. Surprisingly, one study reported 3 rectal NETs with 100% SSTR positivity^41^. Hirofumi et al. reported SSTR2 expression in 10 out of 71 (14.1%) rectal NETs^40^. The low prevalence of SSTR2 expression in previous study may be due to different proportion of high grade (grade 2 and 3) rectal NETs. The present study include 324 (92.6%) cases of grade 1 and 26 (7.4%) cases of grade 2 rectal NETs, while previous study include 51 (71.8%) cases of grade 1 and 20 (28.2%) cases of grade 2 and 3 rectal NETs^40^.

SSTR2 expression in rectal NETs was significantly correlated with favorable clinicopathologic factors, such as small size, absence of lymph node metastasis, low pT classification, low AJCC stage group, and negative chromogranin immunohistochemical expression. In addition, SSTR2 expression was significantly associated with favorable survival and an independent good prognostic factor in rectal NET patients. There have been a few studies of SSTR2 expression and compared their clinicopathologic correlation, including patients’ survival in the pancreatic NETs, but no previous large cohort studies in rectal NETs. SSTR2 expression was significantly correlated with improved survival rate and an independent good prognostic factor in previous studies with pancreatic NETs^26,27,42^, which was consistent with the present study.

To the best of our knowledge, this is the first large-scale study to evaluate the significance of SSTR2 immunohistochemistry in rectal NETs. However, we did not evaluate the value of SSTR2 expressions for SSTR-targeting PET/CT and PRRT in terms of clinical outcomes. Further studies with clinical value of PRRT or imaging modalities using SSTR expression is recommended to assess the overall effects of SSTR expressions in treatment of rectal NET patients.

In conclusion, approximately two-thirds of rectal NETs expressed SSTR2. SSTR2 expression were significantly associated with favorable behavior and good overall survival in patients with rectal NETs. Furthermore, SSTR2 expression can be used as prognostic factors. When metastatic disease occurs, SSTR2 expression can be used a possible target for somatostatin analogues.

## Data Availability

All relevant data are within the manuscript.
